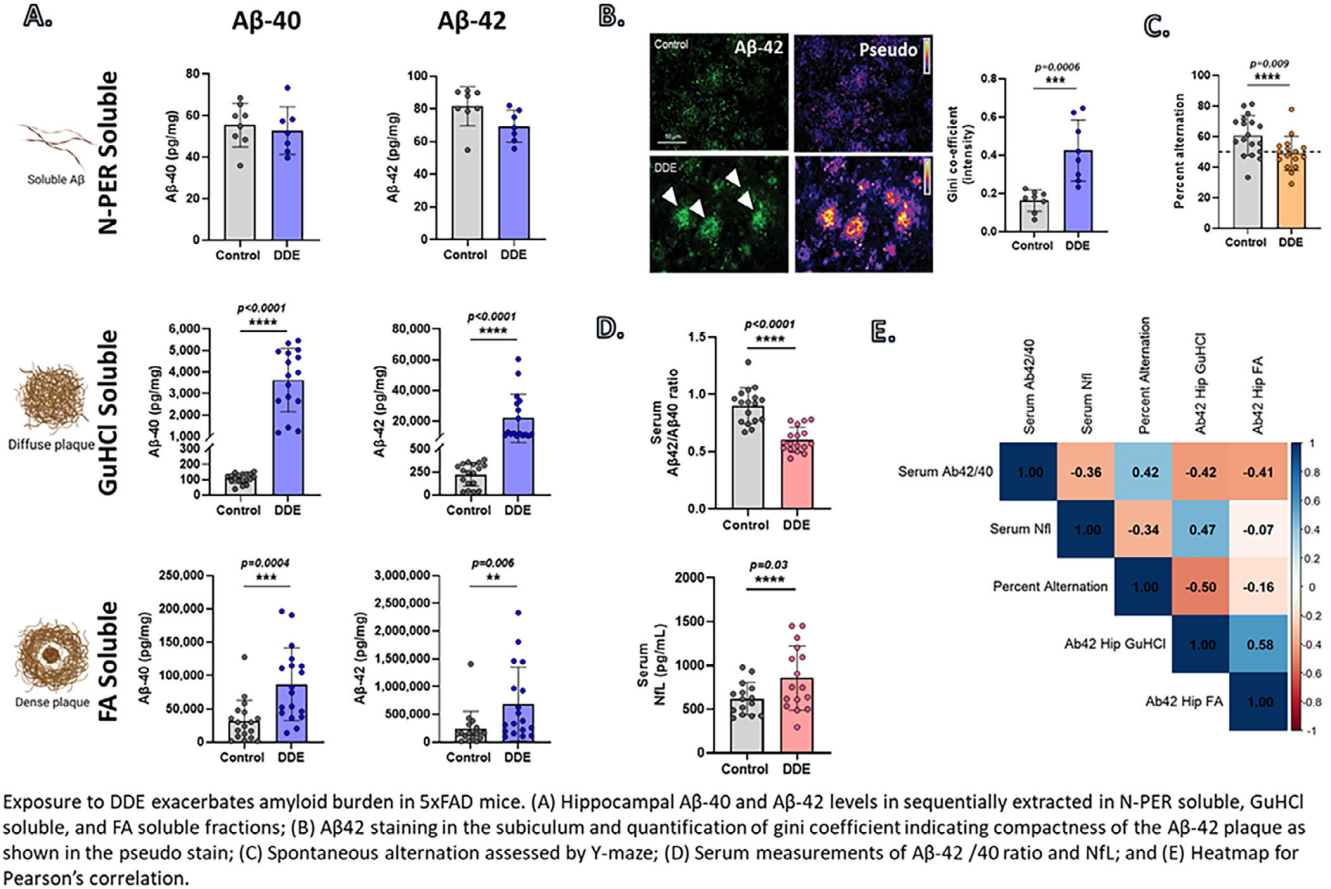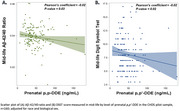# Association of pesticide exposure with plasma Aβ‐42/40 Ratio: Translational evidence from 5xFAD mice and the Child Health and Development Studies

**DOI:** 10.1002/alz70856_106002

**Published:** 2026-01-09

**Authors:** Isha Mhatre‐Winters, Ferass M Sammoura, Piera Cirillo, Arturo J Barahona, Yoonhee Han, Nickilou Krigbaum, Pam Factor‐Litvak, Bruce Link, Young‐Mi Go, Dean P Jones, Barbara Cohn, Jason R Richardson

**Affiliations:** ^1^ Isakson Center for Neurological Disease Research, College of Veterinary Medicine, University of Georgia, Athens, GA, USA; ^2^ Florida International University, Miami, FL, USA; ^3^ Child Health and Development Studies, Public Health Institute, Oakland, CA, USA; ^4^ Robert Stempel College of Public Health and Social Work, Florida International University, Miami, FL, USA; ^5^ Columbia University Mailman School of Public Health, New York, NY, USA; ^6^ University of California ‐ Riverside, Riverside, CA, USA; ^7^ Emory University School of Medicine, Atlanta, GA, USA

## Abstract

**Background:**

Environmental factors, including pesticides, are increasingly associated with an increased risk of Alzheimer's disease (AD). Although banned in 1972, DDT remains persistent due to its long half‐life, bioaccumulation, and residual dumpsites. However ubiquitous, its metabolite, dichlorodiphenyldichloroethylene (DDE), is considered non‐neurotoxic. We previously reported that serum DDE levels were nearly 4x higher in AD patients compared to healthy individuals. Here, we assessed: i) effects of subchronic DDE exposure on serum Aβ‐42/40, brain Aβ pathology, and cognition in 5xFAD mice, and ii) impact of prenatal DDE exposure on midlife plasma Aβ‐42/40 in humans.

**Method:**

Six‐week‐old male 5xFAD mice were exposed to 3 mg/kg *p,p*’‐DDE or corn oil every 3 days for 90 days. At 4.5 months, mice were sacrificed post‐behavioral testing; brains were drop‐fixed for staining, hippocampi dissected, and serum collected for biochemical assays. For prenatal *p,p*’‐DDE exposure, offspring born into the Child Health and Development Studies (CHDS) were recruited in 2010 for follow‐up (∼50 years), completing cognitive tests and providing blood samples. Plasma Aβ‐42/40 ratio in midlife was measured using the Quanterix N3PA kit (*N* = 160).

**Result:**

In DDE‐exposed 5xFAD mice, MSD analysis showed significantly elevated Guanidine‐HCl (GuHCl)‐soluble Aβ42 (∼100‐fold) and formic acid (FA)‐soluble Aβ42 (∼5‐fold), indicating increased insoluble Aβ. Serum Aβ‐42/40 was significantly reduced by 31% in DDE‐exposed mice and was correlated with elevated brain Aβ42 in the GuHCl (*r* = ‐0.42) and FA fractions (*r* = ‐0.40). DDE‐exposed mice displayed working memory deficits, performing 20% worse in Y‐maze, which correlated with low serum Aβ‐42/40 (*r* = 0.42).

In the CHDS cohort, higher prenatal DDE was associated with lower midlife Aβ‐42/40. In logistic models, the Odds Ratio for estimating lower Aβ‐42/40 was 2.6 and 3.2 for DDE tertiles 2 and 3, respectively. The test for trend across DDE tertiles was significant (*p* = 0.04). Higher prenatal DDE was also associated with lower midlife Wechsler Digit Symbol Substitution Task score (*β* = −0.0221).

**Conclusion:**

These findings show that DDE is an active metabolite of DDT and has lasting neurological effects on the amyloid system and cognition. This translational evidence highlights the role of early‐life environmental exposures in AD risk and opportunities for early interventions to prevent disease progression.